# Polar HARP for the polar CMR tagging

**DOI:** 10.1186/1532-429X-14-S1-W15

**Published:** 2012-02-01

**Authors:** Nafiseh Babaee, Abbas N Moghaddam

**Affiliations:** 1 Biomedical Engineering, Tehran Polytechnic, Tehran, Islamic Republic of Iran; 2 Radiology, UCLA, Los Angeles, CA, USA

## Background

The polar coordinate system adapts best to the morphology of the heart. The recently developed sequences that allow the CMR Tagging in the circular and radial directions facilitate the calculation and presentation of the myocardium mechanics. Development of the corresponding processing methods is required to further enhance the utilization of these sequences. Here we suggest a processing method based on the harmonic phase approach to obtain the high-resolution motion in polar coordinate system.

## Methods

**Figure 1:**
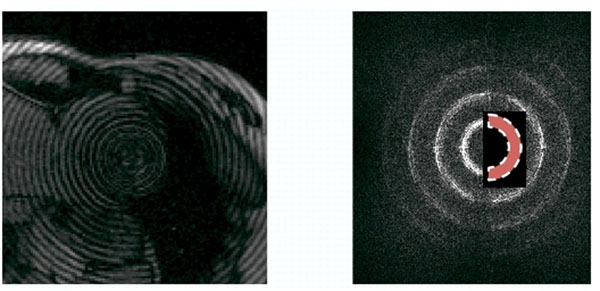
The circularly tagged image of a healthy volunteer and its corresponding k-space. The half circular bandpass filter is shown in the right image.

Short axis polar tagging images were acquired spanning the entire cardiac cycle. Taglines were densely generated for both radial and circular directions with a modified sequence that takes only 10 ms for tagging. Common MR parameters were as follows: 300 mm FOV, 5mm slice thickness, TE/TR = 2.4/44 ms, 250 Hz/pixel, 15° flip angle, 196x196 matrix size. Figure 1 shows one circular tagging image and its corresponding k-space.

Using a half circular bandpass filtering of K-Space, the magnitude and phase images of the fundamental frequency were reconstructed in the circular images. For the radial images a series of narrow bandpass filters were applied for different regions. The harmonic phase images were then unwrapped and adjusted for original and deformed images. Finally the amount of displacement in the direction normal to the taglines was calculated based on the location of pixels with similar phase.

## Results

**Figure 2:**
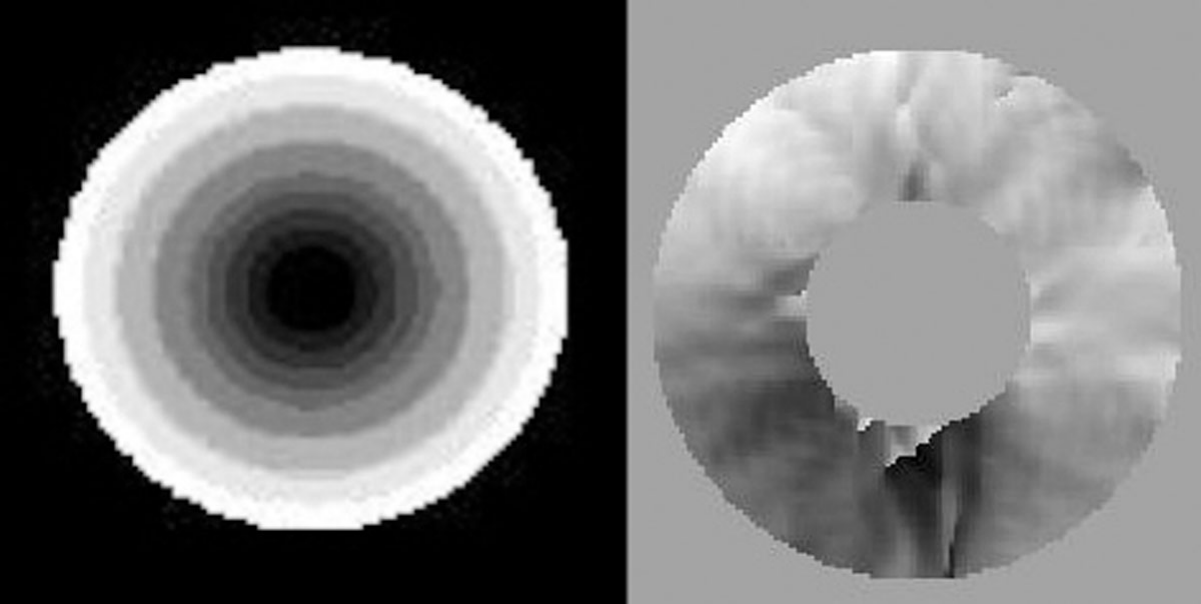
One unwrapped phase image that contains the myocardium in figure 1. The right panel shows the phase difference between the original and deformed images.

Figure 2 shows one actual unwrapped phase image that contains the myocardium in figure 1. In the right panel of this figure the phase difference between the original and deformed images is shown. The results are satisfactory except in a small region in the bottom of the image where the broken taglines corrupted the phase images.

## Conclusions

We have shown that the harmonic phase approach can be utilized in images with densely distributed polar tagging to obtain the high-resolution motion automatically. The technique has wide potential applications in acquired and congenital heart diseases.

